# Conventional androgen deprivation therapy is associated with an increased risk of cardiovascular disease in advanced prostate cancer, a nationwide population-based study

**DOI:** 10.1371/journal.pone.0270292

**Published:** 2022-06-28

**Authors:** Jian-Ri Li, Shian-Shiang Wang, Chuan-Shu Chen, Chen-Li Cheng, Sheng-Chun Hung, Ching-Heng Lin, Kun-Yuan Chiu

**Affiliations:** 1 Division of Urology, Department of Surgery, Taichung Veterans General Hospital, Taichung, Taiwan; 2 Division of the Surgical Intensive Care Unit, Department of Intensive Care, Taichung Veterans General Hospital, Taichung, Taiwan; 3 Institute of Medicine, Chung Shan Medical University, Taichung, Taiwan; 4 Department of Medicine and Nursing, Hungkuang University, Taichung, Taiwan; 5 School of Post Baccalaureate Medicine, National Chung Hsing University, Taichung, Taiwan; 6 Department of Applied Chemistry, National Chi Nan University, Nantou, Taiwan; 7 Department of Medical Research, Taichung Veterans General Hospital, Taichung, Taiwan; Chung Shan Medical University, TAIWAN

## Abstract

**Purpose:**

Androgen Deprivation Therapy (ADT) is the mainstay treatment in advanced prostate cancer. We conducted a nationwide population-based study to evaluate the association of ADT and cardiovascular diseases.

**Methods:**

Between 2005 and 2009, patient data from the National Health Insurance database were obtained. We divided newly diagnosed prostate cancer patients into four groups, injection of gonadotropin-releasing hormone agonists and antagonists, oral antiandrogens, orchiectomy and radical prostatectomy only. Another matched non-cancerous control group was also assigned for comparison purposes. Study outcomes were newly onset Cardiovascular Diseases (CVD) and hospital admissions. Multi-variant Cox proportional regression analysis and the Kaplan–Meier method for cumulative incidence were performed.

**Results:**

A total of 17,147 newly diagnosed prostate cancer patients were found. After exclusion criteria was considered, the 2,565 remaining patients were then divided into 1,088 subjects in the injection group, 286 in the orchiectomy group, 812 in the oral group and 379 in the radical prostatectomy only group. The mean age of all the patients was 71.2 years. Multi-variant analysis showed a significantly increased risk of CVD in the injection group, orchiectomy group, oral group and radical prostatectomy group (HR = 2.94, 95% CI 2.51 to 3.45, p<0.001, HR = 3.43, 95% CI 2.69 to 4.36, p<0.001, HR = 2.87, 95% CI 2.42 to 3.39, p<0.001, HR = 1.93, 95% CI 1.5 to 2.48, p<0.001, respectively). A time dependent increased risk of CVD was also observed amongst the study groups (p<0.001).

**Conclusions:**

ADT is associated with an increased risk of CVD. For long-term prostate cancer castration therapy, doctors should be aware of this complication and arrange for proper management.

## Introduction

Androgen Deprivation Therapy (ADT) is the key treatment in advanced prostate cancer. Since introduction of the first ADT using orchiectomy and estrogen by Huggins and Hodges in 1941, several other regimens, including Gonadotropin-releasing Hormone (GnRH) agonists and antagonists, anti-androgens and androgen receptors inhibitors as well, have become the standards of care in advanced prostate cancer treatment [1,2]. The mainstay of ADT in advanced prostate cancer is continuous androgen blockade, even during the late castration resistant stage; therefore, the longer the advanced prostate cancer patient lives, the longer the duration of ADT treatment is given [3,4]. Although ADT is beneficial in providing better overall survival, a longer ADT treatment duration will lead to increased complications. The most commonly reported adverse events related to ADT were associated with androgen loss, including decreased libido, gynecomastia, weight gain, cognitive deficiency, overactive bladder, metabolic disorders (hyperglycemia, hyperlipidemia, cardiovascular events), and skeletal related events [5–7]. Amongst these cardiovascular events were the major targets in subgroup studies due to their life-threatening nature and consequences. Surgical castration involving an orchiectomy increased the risk of cardiovascular events even higher than did other medical castration medications, as seen in database studies [8,9]. In this study, we used Taiwan’s national health insurance database to stratify the cardiovascular risk amongst prostate cancer patients with or without ADT, while comparing them to a group of non-cancer controls.

## Materials and methods

### Data sources

This study conducted data analysis obtained from the Taiwan National Health Insurance Research Database (NHIRD), which is managed by the Taiwan government. The Taiwan National Health Insurance (NHI) program has covered more than 99% of the population since 1995. In this study, we used systemic data pulled from the Taiwan NHIRD between the years 2005 and 2009. In addition, the study protocols were in accordance to the guidelines, which were approved by the institutional review board of Taichung Veterans General Hospital (CE13151-1). There were no statistically significant differences in either age or healthcare provided between the study group and all enrollees (data not shown). The database contains medical records regarding ambulatory care, inpatient care, and prescription drugs. All the diagnoses were coded according to the International Classification of Disease, Ninth revision, Clinical Modification (ICD-9-CM). In this analysis, access to the NHIRD has been approved by the NHRI Ethics Review Committee.

### Study population and end-point

**Fig 1:**
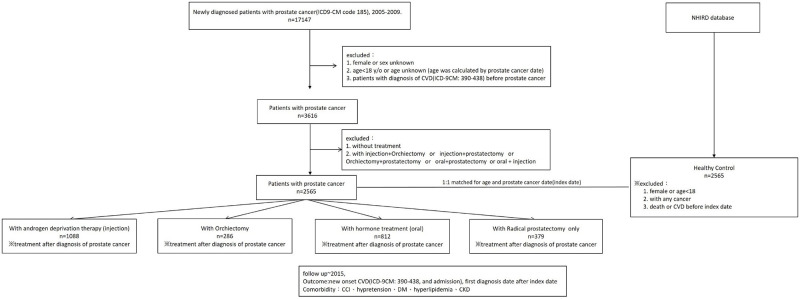
Flow diagram showing the process of Cardiovascular Disease (CVD) patient sampling and participation CVD: Cardiovascular disease; CCI: Charles comorbidity score; DM: Diabetes mellitus; CKD: Chronic kidney disease.

The inclusion criteria of this study groups was newly diagnosed prostate cancer patients with age above 20 between 2005 and 2009 with certain 4 different treatments. These groups included those who had received GnRH agonists or antagonists only (a 2-week combination with anti-androgens was allowed), conventional anti-androgens only (except abiraterone acetate, enzalutamide, apalutamide, darolutamide and other new generation regimens), orchiectomy only, and finally, radical prostatectomy only. There was also an additional group of non-cancer control patients included in the analysis. The health control group was selected between 2005 and 2009 excluding female gender, age less than 18, any pre-existing cancer diagnosis and cardiovascular diseases. All of the subjects in 5 groups were observed to 2016. The different types of ADT were defined as the above-named group divisions using medication codes and operation codes. Fig 1 demonstrates patient selection and sampling algorithm. The study hypothesis is that ADT causes adverse cardiovascular events on prostate cancer patients. The study endpoint was a new onset of cardiovascular disease censored with patient admissions (CVD, ICD-9-CM 390–438, S1 Table) after the prostate cancer index date. Patients included in the study groups must have met two criteria: (i) newly diagnosed prostate cancer between 2005 and 2009; and (ii) receiving of definite treatment as the group defined. The exclusion criteria in the study group were: (i) receiving combination androgen deprivation therapy for more than 2 weeks; (ii) receiving combined radical prostatectomy and androgen deprivation therapy during their lifetime; (iii) having other types of cancer during the study period and (iv) pre-existing CVD including hypertension prior to prostate cancer being diagnosed. The code of prostate cancer was ICD-9-CM 185.0. We also recorded each patient’s Charlson Comorbidity Index score (CCI), hypertension (ICD-9-CM 401–405), diabetes mellitus (DM, ICD-9-CM 250), hyperlipidemia (ICD-9-CM 272), Chronic Kidney Disease status (CKD; ICD-9-CM 582–583) and autoimmune disease (including any one of systemic lupus erythematosis ICD-9-CM 710.0, Sjogren disease ICD-9-CM 710.2 and rheumatoid arthritis ICD-9-CM 714.0) as adjusted factors. The CCI recorded in the study group was based on a one year period prior to prostate cancer being diagnosed. The ratio of all the study group subjects to the control group was 1:1. The matching schema was based upon age and major chronic disease status, such as hypertension, diabetes, hyperlipidemia and chronic kidney disease, which may all be associated with CVD.

### Statistical analysis

The data are presented as the mean values and Standard Deviations (SD) for continuous variables, and proportions for categorical variables. The differences between continuous values were analyzed by using the t-test for continuous variables, and chi-square test for categorical variables. Multivariate Cox proportional hazard regression was used to estimate the Hazard Ratio (HR) and 95% Confidence Interval (CI) for the association between the prevalence of cardiovascular events among the four divided groups. Propensity analysis was used for further confirmation of this association. The cumulative incidence curves were plotted via the Kaplan–Meier method with statistical significance being examined through the log-rank test. All statistical analyses were carried out by SAS software version 9.2 (SAS Institute, Inc., Cary, NC, USA). A p value of <0.05 was considered statistically significant.

## Results

A total of 17,147 subjects in the study group met the primary inclusion criteria. Amongst them, 2,565 cases were selected as study subjects after others were eliminated due to exclusion criteria. The remaining patients included 1,088 subjects placed in the injection group, 286 in the orchiectomy group, 812 in the oral group and 379 in the radical prostatectomy group. The control group also included 2,565 randomly selected subjects from a 1 million non-cancerous population with 1:1 ratio match to the study group (Fig 1).

Table 1 shows the basic characteristics comparison between the study group and healthy controls. The mean age of all the study patient cohorts was 71.2 years. There were no significant differences between the study group and control group in age, hypertension, CKD and autoimmune disease because of the group matching design. In the comparison of comorbidities, a higher CCI score (p<0.001), and higher DM proportion (p<0.001) was seen in the study group when compared with the control group. The censored CVD cases in the control group was 336 (13.1%), which was significantly lower than those in the injection group (318, 29.2%), orchiectomy group (85, 29.7%), oral group (253, 31.2%) and radical prostatectomy group (83, 21.9%) (p<0.001).

**Table 1:** Clinical characteristics of prostate cancer and non-cancerous control subjects.

Variable	Total n = 5,130		Control n = 2,565		Prostate cancer								P-value
					Injection n = 1,088		Orchiectomy n = 286		Oral n = 812		Radical prostatectomy n = 379		
	n	(%)	n	(%)	n	(%)	n	(%)	n	(%)	n	(%)	
Age, years (mean years)	71.2		71.1		71.3								0.537#
Gender													—
Female	0	(0.0)	0	(0.0)	0	(0.0)	0	(0.0)	0	(0.0)	0	(0.0)	
Male	5,130	(100.0)	2,565	(100.0)	1,088	(100.0)	286	(100.0)	812	(100.0)	379	(100.0)	
CCI													<0.001
0	3,931	(76.6)	2,218	(86.5)	736	(67.6)	202	(70.6)	517	(63.7)	258	(68.1)	
1–2	1,035	(20.2)	317	(12.4)	302	(27.8)	72	(25.2)	239	(29.4)	105	(27.7)	
≧3	164	(3.2)	30	(1.2)	50	(4.6)	12	(4.2)	56	(6.9)	16	(4.2)	
Diabetes mellitus													<0.001
No	4,824	(94.0)	2,458	(95.8)	1,006	(92.5)	266	(93.0)	739	(91.0)	355	(93.7)	
Yes	306	(6.0)	107	(4.2)	82	(7.5)	20	(7.0)	73	(9.0)	24	(6.3)	
Hyperlipidemia													<0.001
No	4,869	(94.9)	2,480	(96.7)	1,019	(93.7)	265	(92.7)	758	(93.3)	347	(91.6)	
Yes	261	(5.1)	85	(3.3)	69	(6.3)	21	(7.3)	54	(6.7)	32	(8.4)	
Chronic kidney disease													—
No	5,102	(99.5)	2,554	(99.6)	1,081	(99.4)	283	(99.0)	805	(99.1)	379	(100.0)	
Yes	28	(0.5)	11	(0.4)	7	(0.6)	3	(1.0)	7	(0.9)	0	(0.0)	
Cardiovascular disease													<0.001
No	4,055	(79.0)	2,229	(86.9)	770	(70.8)	201	(70.3)	559	(68.8)	296	(78.1)	
Yes	1,075	(21.0)	336	(13.1)	318	(29.2)	85	(29.7)	253	(31.2)	83	(21.9)	
Autoimmune disease													0.078
No	5092	(92.6)	2554	(99.6)	1075	(98.8)	≥284	(99.3)	804	(99.0)	375	(98.9)	
Yes	38	(7.4)	11	(0.4)	13	(1.2)	≤2	(0.7)	8	(1.0)	4	(1.1)	

CCI: Charlson Comorbidity Index; Note: Autoimmune disease case number in the orchiectomy group was not more than 2 and it could not be confirmed because the regulation of NHIRD.

^#^T test; chi-squared test for all other P-values.

While adjusting for all variables, a significant increased risk of CVD in the injection group, orchiectomy group, oral group and radical prostatectomy group (HR = 2.95, 95% CI 2.51 to 3.45, p<0.001, HR = 3.42, 95% CI 2.69 to 4.36, p<0.001, HR = 2.87, 95% CI 2.42 to 3.39, p<0.001, HR = 1.93, 95% CI 1.5 to 2.48, p<0.001, respectively) was seen when compared with the control group. For single variable impact analysis, increased age, increased CCI and DM were associated with an increased risk of CVD (HR = 1.02, 95% CI 1.01 to 1.02, HR = 1.40, 95% CI 1.2 to 1.62, p<0.001, HR = 1.75, 95% CI 1.31 to 2.34, p<0.001, and HR = 1.31, 95% CI 1.03 to 1.66, p = 0.026, respectively). Results for hyperlipidemia, CKD and autoimmune disease revealed no significant association with CVD risk (Table 2).

**Table 2:** Adjusted risk of developing cardiovascular diseases.

Variable	Adjusted HR	95% CI	P-value
Group			
Control	1.00	—	—
Injection	2.95	(2.51–3.45)	<0.001
Orchiedectomy	3.42	(2.69–4.36)	<0.001
Oral	2.87	(2.42–3.39)	<0.001
Radical prostatectomy	1.93	(1.5–2.48)	<0.001
Age, years (mean±SD)	1.02	(1.01–1.02)	<0.001
CCI			
0	1.00	—	—
1–2	1.40	(1.2–1.62)	<0.001
≧3	1.75	(1.31–2.34)	<0.001
Diabetes			
No	1.00	—	—
Yes	1.31	(1.03–1.66)	0.026
Hyperlipidemia			
No	1.00	—	—
Yes	0.99	(0.77–1.28)	0.945
CKD			
No	1.00	—	—
Yes	1.47	(0.78–2.81)	0.287
Autoimmune diseae			
No	1.00	—	—
Yes	0.78	(0.4–1.5)	0.450

HR was adjusted for all variables in the table.

**Fig 2:**
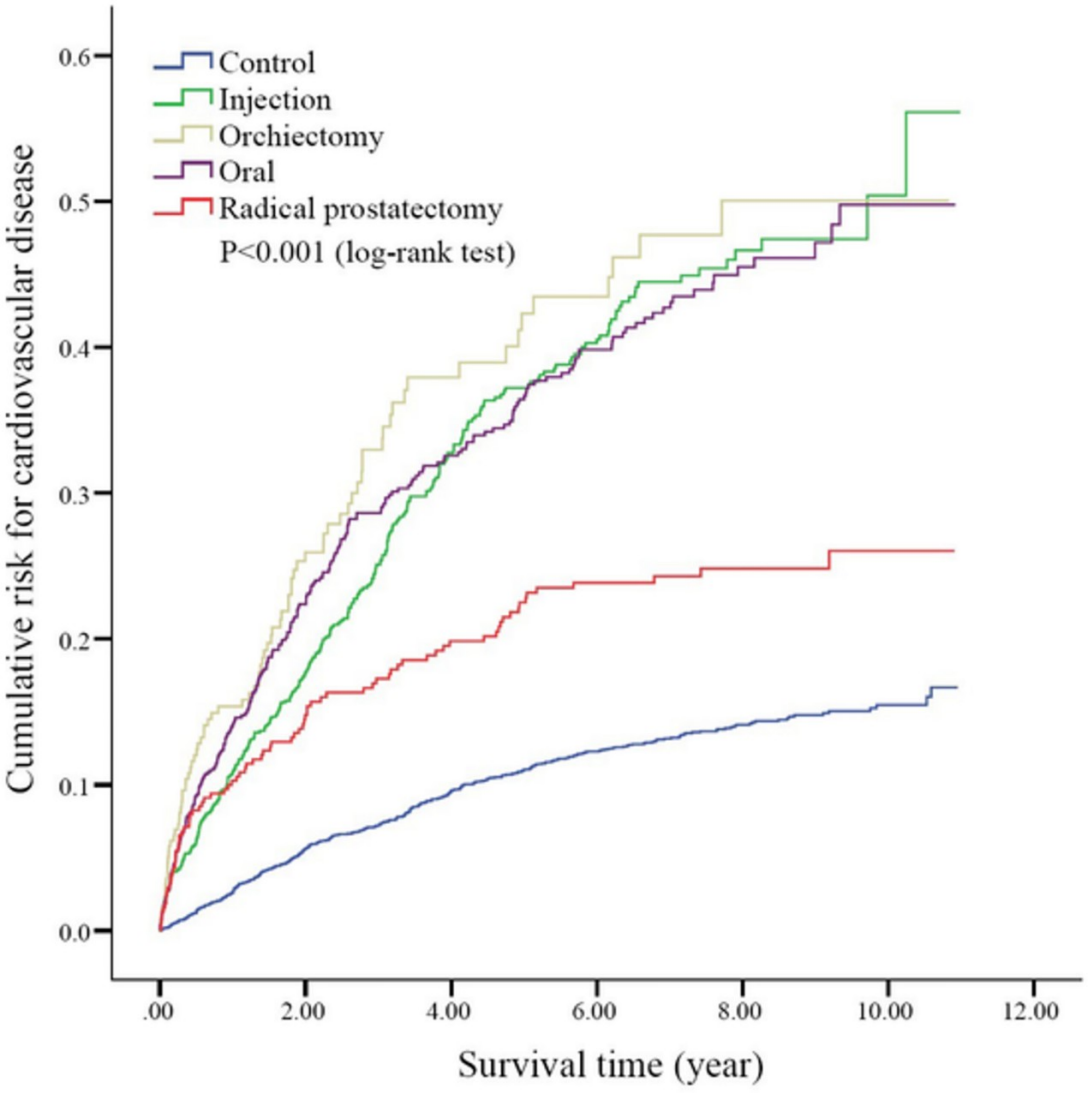
Kaplan-Meier cumulative incidence of developing cardiovascular disease among the five groups (p<0.001, log-rank test) (n = 5,130).

In subgroup analysis, the injection group, orchiectomy group and oral group still showed a higher risk for CVD when compared with the radical prostatectomy group (HR = 1.53, 95% CI 1.19 to 1.96, p = 0.001, HR = 1.78, 95% CI 1.3 to 2.43, p<0.001, and HR = 1.49, 95% CI 1.15 to 1.93, p = 0.001, respectively, Table 3). Regarding analysis of the time sequence pattern, Fig 2 shows a time-dependent, persistent increase of a cumulative incidence of CVD amongst the hormone treatment prostate cancer group, compared with a plateau pattern in the radical prostatectomy and health control groups (Log-Rank test, p<0.0001).

**Table 3:** Adjusted hazard ratio of CVD associated with prostate cancer.

Variable	Adjusted HR	95% CI	P-value
Group			
Radical prostatectomy	1.00	—	—
Injection	1.53	(1.19–1.96)	0.001
Orchiedectomy	1.78	(1.3–2.43)	<0.001
Oral	1.49	(1.15–1.93)	0.003

HR was adjusted for age, CCI and diabetes.

## Discussion

Our population-based study revealed that androgen deprivation, done either medically or surgically, is associated with an increased risk of CVD in advanced prostate cancer patients, while the prostate cancer patients themselves are potentially a risk group for CVD compared with the non-cancerous group. These findings enhance the potential CVD risk prostate cancer patients face without ADT, and even more, the time dependent increasing in CVD risk after long-term ADT.

Along with the application of newly developed novel hormone agents in metastatic Castration Sensitive Prostate Cancer and non-metastatic Castration Resistant Prostate Cancer, these treatment paradigms not only extend the disease control duration but also increase the overall androgen deprivation period [4,10,11]. Therefore, the challenges surrounding advanced prostate cancer treatment has shifted to quality-of-life maintenance, which includes adverse events management during long-term ADT [12–14].

The rationale regarding the association between ADT and end organ adverse events came from consequences stemming from androgen receptor blockade. Androgen receptor mediated metabolic responses act as an important driver towards maintaining both physical and mental health in men [5,15,16]. Several cohort and cross section studies have shown an association between lower serum testosterone (a principal male sex hormone which binds to the androgen receptor) levels and a higher metabolic syndrome percentage and cardiovascular morbidity [17,18]. Alternatively, several randomized control trials have shown that a supplement of testosterone can improve metabolic profiles and possibly decrease the risk of CV morbidity [19].

In order to better understand the real-world association between ADT and CVD, database cohort and meta-analysis studies are used as superior tools in a massive patient population and long follow up duration periods. Keating et al. performed a Surveillance, Epidemiology and End Results (SEER) national cancer registry database survey, and found an association between GnRH agonists use and an increased risk of both coronary heart disease (HR = 1.16, p<0.001) and Myocardial Infarction (MI, HR = 1.11, p = 0.03), amongst newly diagnosed, locoregional prostate cancer patients who were older than 66 years of age. However, men treated with an orchiectomy did not display this association [20]. Saigal et al. used the same database and found a 20% risk increase in CVD amongst 22,816 newly diagnosed, ADT treated prostate cancer patients. The risk of CVD was ADT treatment duration dependent (longer than 12 months), as well as preexisting cardiac disease dependent (twice the risk of CVD than without) [21]. D’Amico et al. then evaluated three randomized trial cohorts, which showed that ADT and an age greater than 65 were both associated with earlier onset fetal myocardial infarction [22]. Our data did reveal some similarity with the above-mentioned series. Nevertheless, we excluded pre-existing confounders and used more precise end-point coding (newly diagnosed cardiovascular events at admission) to eliminate any overestimation, which were steps not taken in most studies. This design not only restricted any overestimation based upon diagnostic codes at outpatient suspicion, but also increased the accuracy of the database and made it more practical for use in a clinical approach. However, based upon this study design, the CVD events which were censored would decrease and compromise subgroup analysis. In addition, patients who received GnRH agonists and antagonists as primary treatment in our series were all in advanced or metastatic disease stages due to regulations in the NHI system as well as in orchiectomy. This baseline design made the included patients more homogenous, while somehow minimizing bias.

Pre-existing comorbidity was also a covariant associated with CVD. Thomsen et al. reported a high association of previous CVD (HR = 2.03, p<0.001), and previous diabetes mellitus (HR = 1.5, p<0.001) with new onset CVD after ADT [23]. Keating et al. declared a relatively elevated risk of CVD (HR = 1.75, p<0.05) when studying previous MI history, but not after ADT [24]. However, there were no differences in any other comorbidities. For patients with comorbidities involving congestive heart disease, peripheral arterial disease, stroke, chronic obstructive pulmonary disease, and chronic renal insufficiency, there were associations with an increased risk of a new onset of MI after ADT. Moreover, chronic rheumatic diseases such as rheumatoid arthritis are considered a risk of cardiovascular diseases because of the background autoimmune effect on vascular endothelium and the treatment side effects [25,26]. However, we did not find significant association between autoimmune diseases and CVD (HR = 0.78, 95% CI = 0.4–1.5, p = 0.45). In this study we used CCI as an indicator of pre-existing comorbidities, which revealed a higher risk for CVD, while there was an independent higher CCI score among all observed subjects, which corresponded to the above series. Hyperlipidemia is a common risk factor for CVD. However, the association of CVD was not significant in our study. It could be the shadow effect of ADT and potential diet behavior change among prostate cancer patients.

Subgroup analysis among different cardiovascular events revealed a controversial association with ADT. Martín-Merino et al. discovered there was an increased risk of coronary heart disease (OR = 4.35), acute myocardial infarction (OR = 3.57), heart failure (OR = 3.19), and hospitalized heart failure (OR = 3.39) when searching through a United Kingdom primary care database [27]. Haque et al. found an elevated risk of cardiac arrhythmia (HR = 1.44) and conducting disorders (HR = 3.11) only in pre-existing CVD patients [28]. Tae et al. showed there was a non-significant difference in cerebrovascular incidences between ADT and non-ADT prostate cancer groups based upon figures taken from a South Korean health care database [29]. Different age categories may also have impact on CVD events. D’Amico et al. found that the ages of 65 years or older were a threshold for experiencing shorter times to fatal myocardial infarction after 6 months of ADT [22]. Smoking is also a well-known risk factor for CVD. Bosco et al. performed a meta-analysis and found that the association between ADT and CVD risk was statistically significant (RR = 1.26) in a 30% smoking status assumption amongst the ADT population [30]. Hershman et al. performed a randomized trial to determine the influence of intermittent ADT on adverse health events and found a 31% increase of ischemic complications using intermittent therapies [31]. Our study did not perform analysis of drug intensity, however, from the possible underestimation of treatments, we still found a high association of an increased CVD risk when comparing the orchiectomy group with the other two control groups. Some clinical trials and meta-analysis have shown GnRH antagonists to have less CVD when compared to with GnRH agonists [32,33]. We did not perform this analysis because the approval for GnRH antagonists was not given until September 2014, which may result in less data being provided in this study. We did not perform subgroup analysis using the above risk categories because of the strict inclusion criteria, resulting in subsequent inadequate study numbers, as well as a lack of smoking status data in the database. However, even without the performance results of proportional hazard assumption tests, we can still trust that the impact of violation is less after following the separated curve between the study groups and two control groups (Fig 2).

Most studies regarding CVD in prostate cancer patients would choose non-ADT, different ADT or an adjusted health group as a control [34]. In this study, we divided our subjects into 3 different ADT groups without any overlap, adding in a localized prostate cancer group who had received radical prostatectomy only, as well as an adjusted healthy non-cancer control group. During subgroup analysis, the orchiectomy group displayed the highest risk (HR = 3.43, 95% CI = 2.69–4.36, p<0.001) of CVD when compared with the healthy control, but analysis did not differ from those in the injection and oral groups. This finding corresponds to previous reports in both the Swedish and SEER database cohort series [23,34]. Furthermore, our newly diagnosed radical prostatectomy only group acted as a baseline prostate cancer control without ADT, which still revealed a potentially higher CVD risk when compared with the healthy control group (HR = 1.93, 95% CI = 1.5–2.48, p<0.001). Previous literature has shown that certain cancers themselves may present specific stereotypes in certain vascular markers or lifestyles which were associated with CVD [35,36]. Our findings also correspond with this hypothesis. Furthermore, we found a plateau pattern of CVD incidence amongst the radical prostatectomy group which may implicate a possible lifestyle medication effect on general health amongst prostate cancer survivors [37].

Finally, we also found a time dependent trend of CVD risk after ADT, which was steeper than that seen in the radical prostatectomy only group and the healthy control group, which was also observed in previous studies [38,39].

Our study has several limitations. First, we used diagnostic and procedure codes to identify subjects in the administration database, in which there may exist some errors, although we applied a similar algorithm reported previously. Second, our oral anti-androgen group may have contained patients at different stages of prostate cancer as compared to the injection and orchiectomy groups because prescription regulation of oral drugs is used more widely in Taiwan. The confounding errors seen in real world practice may influence the results in this group. Third, instead of dividing all the CVD outcomes separately, we only coded them as a single censored end-point. This process therefore does not provide more details of CVD contents among our study populations. Fourth, our study was set prior to the application of new generation hormone agents, which at the time was not compatible with current treatment guidelines. Fifth, we did not conduct an analysis of CVD related mortality, which has been reported as controversial in previous studies [22]. Cause of death data was also not reliable in our database. Instead of CVD mortality figures, CVD hospitalization data may provide a more practical approach for clinicians in the future. Sixth, some pre-existing non-diagnostic confounding factors such as atherosclerosis or atheroembolism which may result in subsequent CVD were not considered in this study setting and can lead to a study bias. Seventh, baseline physical condition and life styles (including cigarette smoking, occupations, exercise and co-medications) may influence the development of CVD and were not exposed in this kind of health insurance database study. These may also deviate the final results. Finally, undiagnosed CVD before inclusion of the study may mislead to the results in this setting and induced an overestimation.

## Conclusion

ADT was associated with an increased risk of CVD among prostate cancer patients. Prostate cancer patients receiving radical prostatectomy only also had higher risk of CVD when compared with non-cancerous healthy control patients. Age and CCI were also independent factors which increase CVD. The ADT duration dependent trend of CVD risk should remind clinicians to pay greater attention to comorbidities in regards to long-term ADT.

## Supporting information

S1 Table ICD-9-CM codes and corresponding diagnosis. (DOCX)

S1 File (XLSX)

S2 File (XLSX)
