# Supplementary material for: Conventional androgen deprivation therapy is associated with an increased risk of cardiovascular disease in advanced prostate cancer, a nationwide population-based study
Source: PLoS One. 2022 Jun 28;17(6):e0270292. doi: 10.1371/journal.pone.0270292 (PMC9239475; doi:10.1371/journal.pone.0270292)
Supplement: S1 Table — (DOCX) [file pone.0270292.s001.docx]

**Supplemental Table 1. ICD-9-CM codes and corresponding diagnosis**

|  | |
| --- | --- |
| ICD-9 code | Diagnosis |
| 390-392 | Acute rheumatic fever |
| 393-398 | Chronic rheumatic heart disease |
| 401-405 | Hypertensive disease |
| 410-414 | Ischemic heart disease |
| 415-417 | Disease of pulmonary circulation |
| 420-429 | Other form of heart disease |
| 430-438 | Cerebrovascular disease |
| ICD-9-CM: International Classification of Diseases; Ninth Revision, Clinical Modification | |
